# Goat Genomic Resources: The Search for Genes Associated with Its Economic Traits

**DOI:** 10.1155/2020/5940205

**Published:** 2020-08-18

**Authors:** A. M. A. M. Zonaed Siddiki, Gous Miah, Md. Sirazul Islam, Mahadia Kumkum, Meheadi Hasan Rumi, Abdul Baten, Mohammad Alamgir Hossain

**Affiliations:** ^1^Genomics Research Group, Faculty of Veterinary Medicine, Chattogram Veterinary and Animal Sciences University (CVASU), Chattogram 4225, Bangladesh; ^2^Department of Genetics and Animal Breeding, Faculty of Veterinary Medicine, Chattogram Veterinary and Animal Sciences University (CVASU), Chattogram 4225, Bangladesh; ^3^Human Biology-Principles of Health and Disease, Ludwig Maximilians University, Munich, Germany; ^4^AgResearch, Palmerston North 4410, Private Bag 11008, New Zealand; ^5^Department of Pathology and Parasitology, Faculty of Veterinary Medicine, Chattogram Veterinary and Animal Sciences University (CVASU), Chattogram 4225, Bangladesh

## Abstract

Goat plays a crucial role in human livelihoods, being a major source of meat, milk, fiber, and hides, particularly under adverse climatic conditions. The goat genomics related to the candidate gene approach is now being used to recognize molecular mechanisms that have different expressions of growth, reproductive, milk, wool, and disease resistance. The appropriate literature on this topic has been reviewed in this article. Several genetic characterization attempts of different goats have reported the existence of genotypic and morphological variations between different goat populations. As a result, different whole-genome sequences along with annotated gene sequences, gene function, and other genomic information of different goats are available in different databases. The main objective of this review is to search the genes associated with economic traits in goats. More than 271 candidate genes have been discovered in goats. Candidate genes influence the physiological pathway, metabolism, and expression of phenotypes. These genes have different functions on economically important traits. Some genes have pleiotropic effect for expression of phenotypic traits. Hence, recognizing candidate genes and their mutations that cause variations in gene expression and phenotype of an economic trait can help breeders look for genetic markers for specific economic traits. The availability of reference whole-genome assembly of goats, annotated genes, and transcriptomics makes comparative genomics a useful tool for systemic genetic upgradation. Identification and characterization of trait-associated sequence variations and gene will provide powerful means to give positive influences for future goat breeding program.

## 1. Introduction

Goats under the genus *Capra* have been raised from the five wild species: *Capra hircus* including *Bezoar*, *Capra ibex*, *Capra caucasica*, *Capra pyrenaica*, and *Capra falconeri* [1]. This evolutionary pattern supports the phenotypic diversity and the high adaptability of the goat to a wide range of environments [2]. Therefore, goats are found to be reared worldwide. Different breeds are usually selected for a different type of product, such as Boer which is for meat, Angora and Cashmere which are for fiber, and Saanen, Toggenburg, and Alpine which are some of the dairy breeds [3]. Black Bengal goat (BBG) is the indigenous breed of Bangladesh famous for its high-quality low-fat meat and skin quality. They have also potential for milk production. Therefore, BBG is considered to be a genetic gold mine for researchers dedicated to improving the economic aspect of goat farming in Bangladesh. In particular, the Black Bengal goat is derived from the wild goat *Bezoar* of *Capra aegagrus* [1]. Any genetic improvement can directly lead to an improvement in its economic traits. On this note, the recent publication of whole-genome assembly and annotated genes of Black Bengal goat [4] has opened possibility more than ever, to identify economic traits with the associated genes using modern cutting edge technology.

Describing a genetic composition of a population requires an understanding of its evolution since genetic variations are the root of all evolution. In an extent, it affects the population's potential to adapt to environmental changes and also affects other phenotypic characteristics. Notably, great quantities of major genes have been identified in goat and sheep populations. These major genes are linked to different reproductive, disease, or production characteristics in the population. The detection of major genes, investigated as candidates to explain the genetic variation of economic traits [5, 6] together with the advance in molecular genetic technologies, opened promising perspectives for improving accuracy, selection intensity, and early selection of the reproducers [7]. Molecular genetics has directed to the detection of individual genes or candidate genes with considerable effects on the traits of economic significance. A candidate gene is a gene that is responsible for a substantial amount of genetic variation of a trait [8]. Candidate gene strategy was proposed employing a direct search for the quantitative trait loci (QTL) [9]. In addition, the genetic variation in the gene influences physiological processes and phenotype expression. Besides, the proportion of genetic and phenotypic variations is likely to influence the reproduction methodology for enhancing important traits in the future. Genetic markers linked with traits of interest can be straightforwardly searched by applying molecular biology techniques. These strategies can recognize genetic variation at specific locations and analyze the relationship between genetic variations at QTL of productive and reproductive traits [10]. The application of molecular genetics for genetic improvement depends on the capacity of genotype individuals for precise genetic loci. The information usefulness of candidate genes in breeding programs has the potential to significantly improve the accuracy of selection and increasing selection differences [3].

Numerous studies have announced that the candidate genes influence growth, milk, reproductive, wool, and disease resistance attributes in goats. The functions of these genes on economically important traits are different. Some genes have synergistic or antagonistic effects in nature for the expression of phenotypic traits [11]. Furthermore, some genes have power over more than one characteristic. For example, the growth hormone (*GH*) gene influences the expression of growth and milk characteristics. The vital candidate genes for goat play a significant role in the production, reproduction, metabolism, sex determination, and disease resistance [4, 12–16]. A study of the candidate genes for major economic traits could be applied for a direct search of QTL in terms of planning future breeding program. A complete, precise goat genome reference provides a vital dimension for advanced genomic selection of productive traits. Substantial progress has been made in goat genome sequencing but still in its infancy compared to other farm animals [15]. Information of several important traits and their respective genes with the knowledge of selection pressure is still unclear [13]. Consequently, the purpose of this review article is to search the reported candidate genes that have an impact on the characteristics of production such as growth, reproductive, milk, wool, and disease resistance in goats. Entirely, these reinforce the opportunity and need for more studies on the inclusion of major genes and large QTLs in genetic and/or genomic assessments in small ruminants.

## 2. Genes Responsible for Economic Traits in Goat

Genes that are related to the economic traits in goats are discussed in details under the different subheadings.

### 2.1. Growth

Traits like growth rate and body weight are among the most economically important traits of meat livestock. In the Nanjiang yellow meat goat breed, the gene *LDB2* (LIM domain-binding factor 2) was identified [13] which is a crucial regulator of transendothelial migration of leukocytes [17]. Another study on domestic goat breeds identified four genes (*TBX15*, *DGCR8*, *CDC25A*, and *RDH16*) responsible for body size [18] (Table 1). The number of mesenchymal precursor cells and chondrocytes is controlled by the *TBX15* gene whereas *DGCR8* is related to osteoclastic development and bone-resorbing activity [19]. In mice, *CDC25A* was found to play essential roles in the myogenic differentiation of myoblasts and G1 quiescence [20]. In adipose tissues of pigs and rats, the *RDH16* gene is found to be involved in energy metabolism [21, 22]. By using WGS analysis in Moroccan goat, the gene *SREBF1* was identified as a critical regulator of lipid homeostasis and *CPT1A* as responsible for the formation of acylcarnitines, a metabolite of fatty acid metabolism [23] which suggests the possible role of this two gene variants in meat fat content. Copy losses of *MYADM* were found in domestic goats [24], and *MYADM* gene family is known to be highly associated with the weight of weaned lamb and erythrocyte morphology [25]. A recent study reported the association of *MYTIL* (myelin factor 1-like), an early-onset obesity-related gene [26]; *APOL3* (apolipoprotein L3), lipid-transport and metabolism-associated genes; and *STIM1* (stromal interaction molecule 1) involved in catty body weight gain [12]. Other meat breed trait-associated genes (TAGs) found in elite goat breeds are *HMGXB3* and *SLC26A2* [12]. The other candidate genes related to growth traits are *GH*, *GHR*, *IGF-1*, *LEP*, *POU1F1*, *MSTN*, and *BMP* [11]. The identification of TAGs in the BBG population can open a new window to improve its meat quality.

### 2.2. Milk Production

**Figure 1:**
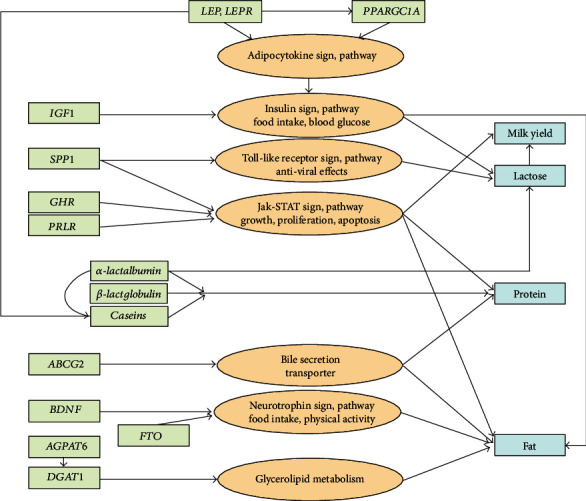
Overview of pathways for major genes involved in the production of milk. Green boxes are genes, orange circles are the pathways the genes are involved in, and blue boxes are the milk production traits that are affected (adapted from [33]).

Though the quantity of milk and milk fat and proteins is a particularly essential trait in dairy livestock, little is known about the regions of the genome that influence these traits in goats [28] and very few positive associations of any allele with milk characteristics were so far reported. A recent analysis on trait-associated genes (TAGs) of domestic goat breeds reported the association of *RPL3* (ribosomal protein L3) by regulating energy balance during lactation work and the association of *VPS13* (vacuolar protein sorting 13) family with goat milk production [12] as the previous association was found in several farm animals. Specifically, *VPS13B* (homolog B) was found to be associated with leg morphology, related to fertility and milk production in cattle and buffalo [29], and *VPS13C* (homolog C) is suggested to act on glucose homeostasis for high milk production in dairy cows [30]. From the CNV analysis of goat breeds, the gene *BTNA1* was found to be essential for secretion of milk-lipid droplets [27] (Figure 1). Another study implies that overexpression of growth hormone (*GH1*) in transgenic goats may stimulate breast development and enhances milk production by modulating alveolar cell proliferation or branching through the MAPK signalling pathway and SNPs in *GH1* were found to be associated with milk production in dairy cows [31]. One of the most intensively analyzed genes is milk protein genes, where significant effects were assessed for the *α*s1-casein, in both sheep and goats [8]. In the Sarda goat breed, genetic polymorphism within the casein genes *CSN1S1*, *CSN2*, *CSN1S2*, and *CSN3* gene loci was investigated, and *CSN2* and *CSN1S2* genotypes were found to affect milk protein content [33]. Candidate genes that might be involved in goat milk production according to their function (Figure 1) include *LEP*, *LEPR*, *IGF1*, *GHR*, *PRLR*, *AGPAT6*, and *DGAT1* [33] (Table 2).

### 2.3. Prolificacy/Litter Size

The continuous attempt of animal geneticists' to maximize profit from livestock can be accomplished by improving the genetic potential using suitable selection methods. For attaining maximum benefit from livestock, genetic selection is a quintessential tool. Livestock with improved reproductive competence and increased fertility rate will eventually pave the way for the economic gain of farmers [50]. Litter size (LS) is a deciding and complex economic attribute within the goat industry. Multiple genes and factors [51] involved in ovarian follicular development, oocyte maturation, ovulation, fertilization, embryogenesis, embryo implantation, and uterine receptivity additively appear to control litter size [52]. A study in high fecundity dairy goats reveals the positive association of several genes including *SMAD2*, *ADCY1*, *CCNB2*, *AR*, *DNMT3B*, *AMHR2*, *ERBB2*, *FGFR1*, *MAP3K12*, and *THEM4* [53] (Table 3). Normal fertile oogenesis and some ovulatory processes of a female are maintained by SMAD2 protein, a member of the TGF-beta superfamily [54]. In oocyte meiotic arrest and resumption, another candidate gene *ADCY1* (adenylate cyclase 1) helps to cyclize AMP to form cAMP [53]. The *CCNB2* (cyclin B2) gene is known to activate *Cdk1* (cyclin-dependent kinase1) in oocytes, and nullizygous mutation in it was reported to decrease litter size in mice [55, 56].

An androgen receptor (AR) is a hormone-inducible DNA-binding transcription factor that plays an essential role in reproduction by transmitting androgen signals. Moreover, *AR* knocked out female mice display a lower number of pups per litter [57] and male reported with severe impairments in reproductive tracts and sexual behavior [58]. A member of DNA methyltransferases (*DNMTs*), *DNMT3B* appears to play a role in human preimplantation embryo development by participating in global DNA methylation [59]. The identified reproduction-related gene *AMHR2* (anti-Mullerian hormone receptor, type II) is a member of protein family AMHR which is a negative regulator of ovulation and dysfunction of it leads to anovulation in humans [60]. *ERBB2* (Erb-b2 receptor tyrosine kinase 4) is a steroid hormone receptor and involved in the several physiological mechanisms including calcium signalling pathway [61]. Among other candidate genes, *THEM4* (thioesterase superfamily member 4) is involved in the PI3k-Akt signalling pathway; *FGFR1* (fibroblast growth factor receptor 1) and *MAP3K12* (mitogen-activated protein kinase kinase kinase 12) are involved in the MAPK signalling pathway which is a downstream regulator of several genes, but their exact role in reproduction is not known yet [53]. Moreover, that genetic variation like nonsynonymous exonic SNPs in *SETDB2* (SET domain, bifurcated 2) and *CDH26* (cadherin 26) by being colocalized in selected regions may take part in fecundity traits in dairy goats [53]. In another study on Meigu goat, the association of *KHDRBS2* (KH RNA binding domain containing, signal transduction associated 2) gene has been identified [13]. Genetic variations in this gene were associated with the number of teats in White pigs [62] and pregnancy status in Brahman beef cattle [63]. Bone Morphogenetic Protein 15 (*BMP15*), a member of the oocyte, secreted protein BMP, the largest subgroup of the transforming growth factor-beta (TGF-*ß*) superfamily [64] stimulates follicle growth, granulosa cell proliferation, and cell survival signalling [65]. Genetic variations in *BMP15* (also known as *FecX*) gene were reported to be associated with increased ovulation rate and litter size in sheep [66] and positive association with triplets also found in Guizhou goat population [67]. However, such polymorphism associations were not found in Indian (Marwari) [68] and Chinese [69] goat breeds. Bone Morphogenetic Protein Receptor 1B (*BMPR1B*) or Booroola fecundity gene (*FecB)* plays an important role in increasing ovulation rate and litter size. The exonic (exons 1, 2 ,and 6 to 9) and promoter regions of the *BMPR1B* gene with genetic variations were found in Black Bengal goat [70], and another eight indigenous goat breed polymorphisms were also identified [71]. In the female reproduction of mammals; control of cell division, ovarian folliculogenesis, oogenesis, and secretory activities are maintained by growth hormone (*GH*) [72–74] and polymorphisms in this gene were reported to have an effect on litter size in Boer and Matou goats [75]. The other previously identified polymorphic genes associated with litter size are *KISS* (kisspeptin in goat), *GDF9* (growth differentiation 9 in Indian goat), *POUF1* (pituitary transcription factor-1 in goat), *PRLR* (prolactin receptor in Chinese black goat), *GPR54* (kisspeptin receptor 54 in goat), *IGF1* (insulin-like growth factor 1 in Black goat), and *FSHR* (follicle-stimulating hormone receptor in Boer goat) [50]. In the high fecundity group, copy number variation (duplicated) was found for Prolactin-related protein 1 and 6 (PRP1 and PRP6) regulating reproductive processes [76]. Considering reproductive traits are polygenic, identification of all the available genetic variations of the goat population will help to carry out the marker-assisted selection with improved prolificacy in a more precise manner.

### 2.4. Disease Resistance

Diseases are the main impediment of productivity of the goat in most tropical countries [77]. An effective management program to keep goats healthy is necessary for production. There have been few selection experiments or breeding programs for disease resistance in goats; however, there is evidence for both within- and between-breed variations which can be utilized in breeding programs [78]. The common diseases which affect goats in tropical countries are scrapie, peste des petits ruminants (PPR), helminthiasis, contagious ecthyma, fever, pox, pneumonia, anthrax, ectoparasite, alopecia, anorexia, etc. The occurrence of scrapie, a fatal neurodegenerative disease, is strongly influenced by alterations in host goat gene encoding prion protein (*PrP*) [79]. Peste des petits ruminants (PPR) is one of the most challenging factors for goat husbandry in the region due to high mortality of goats with this viral disease, leading to the massive economic loss to marginal and landless farmers [80]. The annual economic loss in India due to PPR disease of goats is estimated at 843.53 million US $ [81]. In 2015, a study on goat transcriptome reported about the dysregulation of immunoregulatory pathways and genes encoding transcription factors (*TF*s) in case of PPR viral infection [82]. The association of tripartite motif protein (TRIM56) and interferon regulatory factor (IRF4/5) protein act by restricting PPR virus replication was identified [82] (Table 4). The protein TRIM56 was also found to restrict bovine viral diarrhea by inducing interferon-stimulated genes (*ISGs*) that are transcriptionally regulated by *IRF* genes [83]. Another candidate gene is *MHCI* (major histocompatibility complex, class I) whose polymorphisms had been often reported to be involved in the resistance/susceptibility to a variety of infectious and parasitic diseases in ruminant species [84]. Interestingly, in one comparative study between wild and domestic goat, four deleted gene copies (*ABCC4*, *PRAME*, *CD163L1*, and *KIR3DL1*) and two gained gene copies (*CFH* and *TRIM5*) were found in domestic goats involved with the immune system [27]. Natural resistance capability of Black Bengal goats (BBG) is promising which makes them less susceptible to some sorts of diseases but PPR. In breeding animals for enhanced resistance to the disease, the information about heritable differences between animals is vital [78]. However, so far, in the state of knowledge, no molecular genetic approach has been yet applied to identify the genetic basis of disease resistance capacity of BBG.

### 2.5. Adaptation

Among the most critical environmental challenges to envisage animals is the low oxygen availability of high-altitude regions which causes hypoxia, imposes severe constraints on aerobic metabolism, and leads to high-altitude illness [85, 86]. In recent years, thus, the mechanisms of hypoxic adaptation have become of great interest. Identification of selection signatures of high altitude adaptation has been performed across a wide range of species, including humans [87], goat [18], cattle [88], and chicken [89] by genome-wide scans or whole-genome resequencing analysis. To elucidate the adaptive mechanism process in goat is essential for future research to have bred with high adaptive capability. By analyzing whole-genome sequencing of eight goat populations in Tibetan goat (China), the genes identified for hypoxic adaptation were *CDK2*, *SOCS2*, *NOXA1*, and *ENPEP* [18]. *CDK2* is involved in hypoxia-induced apoptosis in cardiomyocytes [90], *SOCS2* is found as a selective gene in Tibetan sheep [91], *NOXA1* by being an activator of *NOX1*-mediated HIF1 (hypoxia-induced factors) response is associated with intermittent hypoxia conditions [92], and *ENPEP* is also a candidate gene of high altitude adaptation in Andeans [93]. In a recent study in cashmere goat, *EPAS1* (encompassing endothelial PAS domain protein 1) gene was found as a possible gene associated with high-altitude adaptation [13] and the association of this gene was also found in humans [94] and dogs [95] to a low oxygen environment. In another two recent studies on sheep, several candidate genes of high altitude adaptation such as *IFNGR2*, *MAPK4*, *NOX4*, *SLC2A4*, *PDK1* [96], *IDE*, *IGF1*, *P2RX3*, *PHF6*, *PROX1*, and *RYR1* were also reported [97] (Table 5). Under normal oxygen concentrations, oxygen-dependent enzymes give signalling for the degradation of the transcription factors of HIF. When oxygen levels fall, enzyme activity reduces and HIF remains intact in the cell and promotes the transcription of genes that help the cell cope with the low oxygen conditions [98].

To live in a cold and dry environment, goat gains some adaptive features like fine cashmere fibers which help to combat heat loss [99]. Other physiological mechanisms also evolved to maintain mineral and energy homeostasis [18, 24]. For example, in cultured adrenal cells, adenylyl cyclase (AC) stimulates cAMP which is involved in cAMP-induced cell proliferation and a key mediator of Na and water transport. Another gene, *ADCY4* (adenylyl cyclase 4), was also found to be involved in the regulation of the insulin secretion, adrenergic signalling in cardiomyocytes, rap1 signalling pathway, cGMP-PKG signalling pathway, and oxytocin signalling pathway [100, 101]. Besides that, four genes *ROCK1* (Rho-associated protein kinase 1), *ACNA1C* (calcium voltage-gated channel subunit alpha1 C), and *OXTR* (oxytocin receptor) were also involved in the oxytocin signalling pathway which functionally related to the regulation of skin development, fat metabolism, and ion homeostasis [99]. Most importantly, *SLC24A4* (sodium/potassium/calcium exchanger 4) by being located in the classical HIF-1 (hypoxia-induced factors) pathway plays a central role in hypoxia-related cellular responses [96]. Among other candidate genes, *CACNA2D1* (calcium channel, voltage-dependent, alpha2/delta subunit 1), *AGT* (angiotensinogen), and *PTGER2* (prostaglandin E receptor 2) were involved in the renin secretion pathway and may also play essential roles in hypoxia-mediated cellular responses [99].

### 2.6. Coat Color

By using comparative population, genomic analysis in six phenotypically diverse goat breeds from pooled whole-genome resequencing data, *IRF4*, *EXOC2*, *RALY*, *EIF2F2*, and *KITLG* genes, were found within the selection signals for coat colors [13] (Table 6). Single nucleotide polymorphisms (SNPs) in interferon regulatory factor 4 (*IRF4*) and exocyst complex component 2 (*EXOC2*) previously reported being associated with skin pigmentation, hair color, or skin sensitivity to the sun in humans by enhancing melanin synthesis by upregulating the expression of tyrosinase [103–105]. Moreover, in another genome-wide association study (GWAS), it was also reported that variations in *EXOC2* are associated with tanning ability [106] which indicates that this gene might be associated with coat color. RALY heterogenous nuclear ribonuclear protein (*RALY*) and eukaryotic translation initiation factor 2 subunit 2 (*EIF2S2*) influence the skin and hair pigmentation through another goat gene *ASIP*, which encodes agouti signalling proteins that promote hair follicle melanocytes to synthesize pheomelanin in animals [107]. High-frequency domestication-specific copy number variations (CNVs) such as *ASIP* and *AHCY* genes are related to skin color in elite goats (*Capra* species) [12], which is consistent with previous findings in sheep [108]. The *KITLG* (KIT ligand) gene is involved with the migration of melanocytes [102] and found as a selection signature in Taihang black goat [13]. Another study on Taihang black goat reported about six overlapped loci with candidate genes (*ASIP*, *KITLG*, *MSANTD1*, *HTT, GNA11*, and *DST*) [18]. The locus with *MSANTD1* and *HTT* was also identified as the strongest selective sweeps in European black goat population [23], thereby highlighting the importance of this locus in the determination of black coat color in goats. By using RNA-sequencing technique, differentially expressed mRNAs and lncRNAs have been identified, whose main activities were in the *cis-* and *trans*-configuration of proteins involved in melanin biosynthesis, melanocyte differentiation, developmental pigmentation, and melanosome transport [109]. According to color gene database, in the other CNV regions, the candidate genes *ATRN*, *GNAQ*, *HELLS*, *MUTED*, *OSTM1*, *TRPM7*, *VPS33A*, Adamts20, *MITF*, *OCA2*, and *SLC7A11* are also associated with coloration.

### 2.7. Cashmere Fiber

Cashmere goat grows an outer coat of coarse hairs from its primary hair follicles and an inner coat of fine wool from its secondary hair follicles. This exclusive fine wool fiber is known as cashmere wool or cashmere [110]. It is softer and finer than sheep's wool and contributes high economic values to the textile industry and impoverished remote areas [111]. In mammals, coat hair acts as a protective material against environmental changes. Unlike other mammals, cashmere-producing goats have a double coat consisting of the outer coarse hair produced by primary hair follicles (PHF) and the inner fine coat (cashmere) produced by secondary hair follicles (SHF). By analyzing the sequence, heterozygosity and divergence of a well-known cashmere goat breed, Inner Mongolia cashmere, with other goat breed regions encompassing the *LHX2*, *FGF9* (fibroblast growth factor 9) and *WNT2* genes were found to be associated with cashmere fiber traits [18] (Table 7). *LHX2* is involved with the development of SHF [111], WNT2 involved in hair follicle initiation [112], and *FGF9* can promote hair follicle regeneration after wounding [113]. From resequencing genome data of cashmere breeds, another finding suggested that selected genome regions with genes (*FGF5*, *SGK3*, *IGFBP7*, *OXTR*, and *ROCK1*) are potentially involved with cashmere fiber traits. *FGF5* regulates hair length, and disruption of this gene in cashmere goat leads to more secondary hair follicles and longer fiber [114], but *SGK3* (serum/glucocorticoid-regulated kinase 3) plays an important role in the development of postnatal hair follicle [115].

## 3. Future Prospects

Advances in molecular genetic techniques may provide an option to enhance the genetic advancement of a goat. Numerous techniques have been developed to clarify the mechanisms concerned in phenotypic expression at the DNA level. The growth of next-generation molecular tools to recognize genomic genetic variants has made it conceivable to apply whole-genome scanning techniques, genome-wide association studies, and genomic selection to improve various characteristics in goat [116]. Evidence has demonstrated that the use of genomic information to select goat creates the prospect to enhance genetic gains. With the availability of whole-genome sequencing technology, the information of different goat genomes is becoming more available. On the other hand, high-throughput RNA sequencing provides a powerful tool for profiling the transcriptome and detecting gene expression in given cells or tissues, identifying differentially expressed genes (DEGs) and novel transcripts. These techniques have been successfully used for genome-wide analysis of mRNAs in multiple organisms including bacteria to mammals. Remarkable surges mainly owing to the introduction of the CRISPR-Cas tools in 2012 have begun a stir in genome editing technologies but now are used efficiently to alter the genome of organisms with customizable specificities to attain fancied benefits. Till now, by using the CRISPR-Cas9 tool, several successful attempts of producing transgenic livestock like a pig, cattle, sheep, and chicken with disease resistance capacity as well as with improved traits have been reported. To improve the economic traits and overcome disease susceptibility of goat, precise, versatile genome editing tools like CRISPR-Cas9 can be new hope for improved transgenic goat development. However, these technologies are underutilized in goats and more multidisciplinary research in this field should be carried out.

## Figures and Tables

**Table 1:** Genes that are related to growth traits in goat.

Gene name	Chromosome	Exon	Function	Founder breeds/population	References
*LDB2*	6	9	Regulator of transendothelial migration of leukocytes	Nanjiang yellow goat, Jinhai yellow chicken	[13, 17]
*TBX15*	3	9	Responsible for body size & controlling the number of mesenchymal precursor cells and chondrocytes	Guizhou Small goat	[18, 19]
*DGCR8*	—	—	Responsible for body size, related to osteoclastic development and bone-resorbing activity	Guizhou Small goat	
*CDC25A*	22	15	Responsible for body size and involved in the myogenic differentiation of myoblasts and G1 quiescence	Guizhou Small goat	[18, 20]
*RDH16*	5	4	Involved in energy metabolism	Guizhou Small goat	[18, 21, 22]
*SREBF1*	19	8	Critical regulator of lipid homeostasis	Moroccan goat	[23]
*CPT1A*	29	19	Responsible for the formation of acylcarnitines, a metabolite of fatty acid metabolism	Moroccan goat	
*MYADM*	18	1	Associated with the weight of weaned lamb and erythrocyte morphology	Columbia sheep, Polypay sheep, Rambouillet sheep, Bamu wild goat, Khonj wild goat, Australian feral Rangeland goats, Boer goats, Australian cashmere goat	[25, 27]
*APOL3*	—	—	In lipid transport and metabolism	Leizhou goat	[12]
*STIM1*	15	14	Involved in catty body weight gain	Leizhou goat	
*HMGXB3*	7	21	Meat breeds trait-associated genes of elite goat breeds	Leizhou goat	
*SLC26A2*	7	9			
*GH*	19	5	Related to the growth of the body	Thai Native, Anglo-Nubian, Boer, and Saanen goat	[11]
*GHR*	20	13			
*IGF-1*	5	7			
*LEP*	4	3			
*POU1F1*	1	6			
*MSTN*	2	3			
*BMP*	16	10			

**Table 2:** Genes that are related to milk production traits in goat.

Gene name	Chromosome	Exon	Function	Founder breeds/population	References
*LEPR*	3	22	Impact upon blood glucose regulation, milk yield, and milk fat production	Holstein dairy	[34]
*LEP*	4	3			[33–35]
*BDNF*	15	6	Impact upon food intake and thus nutrient and energy availability for milk production	Holstein dairy	[31, 36–39]
*FTO*	18	9			
*IGF1*	5	7			
*ABCG2*	6	22			
*GHR*	20	13	Affect growth, proliferation, and apoptosis of cells	Holstein dairy, Ayrshire dairy, Chinese cattle	[34, 40–43]
*PRLR*	20	11			
*DGAT1*	14	18	Involved directly in triglyceride (milk fat) synthesis	Xinong Saanen and Guanzhong goat	[44–47]
*AGPAT6*	27	14			
*RPL3*	5	10	Regulating energy balance during lactation work	Saanen goat	[12]
*VPS13*	8	73	Associated with milk production	Saanen goat	
*VPS13B*	14	65	Associated with leg morphology, related to fertility and milk production	Holstein dairy	[29]
*VPS13C*	10	85	Act on glucose homeostasis for high milk production	Holstein dairy	[30]
*OPN*	6	7	Milk yield and milk fat production	Holstein dairy	[41, 48]
*BTNA1*	23	9	Essential for the secretion of milk-lipid droplets	Bamu wild goat, Khonj wild goat, Australian feral rangeland goat, Boer goat, Australian cashmere goat	[27]
*GH1*	19	5	May stimulate breast development and enhances milk production and associated with milk production	Holstein dairy	[31]
*αs1-casein*	6	19	Major milk protein production and milk formulation	Vallesana, Roccaverano, Maltese, Jonica, and Garganica goat	[8]
*CSN1S2*	6	19	Encode the major fraction of milk proteins	Sarda goat	[32, 49]
*CSN2*	6	9			

**Table 3:** Genes that are related to prolificacy/litter size trait in goat.

Gene name	Chromosome	Exon	Function	Founder breeds/population	References
*SMAD2*	24	12	Maintain normal fertile oogenesis and some ovulatory processes of a female	Laoshan dairy goat	[53, 54]
*ADCY1*	4	20	Involved in oocyte meiotic arrest and resumption	Guizhou Small goat, Laoshan dairy goat	[18, 53]
*CCNB2*	10	9	Activate *Cdk1* (cyclin-dependent kinase1) in oocytes	Laoshan dairy goat	[53, 55, 56]
*AR*	X	8	Plays an essential role in reproduction by transmitting androgen signals	Laoshan dairy goat	[53, 57, 58]
*DNMT3B*	13	22	Play a role in preimplantation embryo development	Laoshan dairy goat	[53]
*AMHR2*	5	11	Works as a negative regulator of ovulation		
*ERBB2*	19	27	Involved in the several physiological mechanisms including calcium signalling pathway		
*FGFR1*	27	20	Involved in the MAPK signalling pathway. May take part in fecundity traits in dairy goats		
*MAP3K12*	5	16			
*THEM4*	3	6			
*SETDB2*	12	14			
*CDH26*	13	16			
*KHDRBS2*	23	9	Associated with pregnancy status	Meigu goat	[13, 62]
*BMP15*	X	2	Stimulates follicle growth, granulosa cell proliferation, and cell survival signalling	Guizhou goat	[64–67]
*BMPR1B or FecB*	6	20	Plays an important role in increasing ovulation rate and litter size	Black Bengal, Boer, and Matou goat	[70, 71, 75]
*KISS*	16	2	Associated with a litter size of the goat reproduction system	Indian goat, Chinese black goat, and Boer goat	[50]
*GDF9*	7	3			
*POUF1*	1	6			
*PRLR*	20	11			
*GPR54*	7	5			
*IGF1*	5	7			
*FSHR*	11	10			
*PRP 1*	18	6	Regulation of reproductive processes		
*PRP 6*	18	5			

**Table 4:** Genes related to disease resistance in goat.

Gene name	Chromosome	Exon	Function	Founder breeds/population	References
*TRIM56*	25	3	Restricts PPR virus replication and bovine viral diarrhea by inducing interferon-stimulated genes	Indian native goat	[82, 83]
*IRF4/5*	23	9	Restricts PPR virus replication		
*ABCC4*	12	31	Involved with the immune system	Bamu wild goat, Khonj wild goat, Australian feral Rangeland goat, Boer goat, Australian cashmere goat	[27]
*PRAME*	Un	5			
*CFH*	16	22			
*TRIM5*	15	8			

**Table 5:** Genes related to adaptation in goat.

Gene name	Chromosome	Exon	Function	Founder breeds/population	References
*CDK2*	5	8	Involved in hypoxic adaptation	Tibetan cashmere goat	[18, 89–93]
*SOCS2*	5	6			
*NOXA1*	11	14			
*ENPEP*	6	20			
*EPAS1*	11	16	Associated with high-altitude adaptation		[13, 97, 102]
*IFNGR2*	1	10			
*MAPK4*	24	6			
*NOX4*	29	18			
*SLC2A4*	19	11			
*PDK1*	2	11			
*IDE*	26	26			
*IGF1*	5	7			
*P2RX3*	15	12			
*PHF6*	X	11			
*PROX1*	16	7			
*RYR1*	18	106			
*ADCY4*	10	26	Involved in the regulation of the insulin secretion, adrenergic signalling in the various pathways	Inner Mongolia and Liaoning cashmere goat	[100, 101]
*ROCK1*	24	33	Involved in the oxytocin signalling pathway which functionally related to the regulation of skin development, fat metabolism, and ion homeostasis		[99]
*ACNA1C*	5	50			
*OXTR*	22	5			
*SLC24A4*	21	17	Plays a central role in hypoxia-related cellular responses		
*CACNA2D1*	4	40	Involved in the renin secretion pathway and may also play essential roles in hypoxia-mediated cellular responses		
*AGT*	28	5			
*PTGER2*	10	2			

**Table 6:** Genes that are related to coat colors in goat.

Gene name	Chromosome	Exon	Function	Founder breeds/population	References
*IRF4*	23	9	Associated with skin pigmentation, hair color, or skin sensitivity	Nanjiang yellow goat, Taihang black goat	[13, 103–106]
*EXOC2*	23	29			
*RALY*	13	10	Influences the skin and hair pigmentation	Nanjiang yellow goat, Taihang black goat	[13, 107]
*EIF2S2*	13	9			
*ASIP*	13	5	Encodes agouti signalling proteins that promote hair follicle melanocytes to synthesize pheomelanin in animals	Taihang black goat, Saanen, Liaoning cashmere, and Leizhou goat	[12, 18, 107]
*KITLG*	5	10	Involved with the migration of melanocytes	Taihang black goat	[13, 89, 102]
*AHCY*	13	10	Related to skin color in elite goats	Saanen, Liaoning cashmere, and Leizhou goat	[12]
*MSANTD1*	Un	5	Determination of black coat color in goats	Taihang black goat, European black goat	[18, 23]
*HTT*	6	56			
*GNA11*	7	7			
*DST*	23	111			
*ATRN*	13	30	Associated with coloration of the coat	Bamu wild goat, Khonj wild goat, Australian feral Rangeland goats, Boer goats, Australian cashmere goat	[27, 65, 109]
*GNAQ*	8	7			
*HELLS*	26	22			
*OSTM1*	9	6			
*TRPM7*	10	39			
*VPS33A*	17	13			
*Adamts20*	5	39			
*MITF*	22	15			
*OCA2*	2	24			
*SLC7A11*	17	13			

**Table 7:** Genes related to cashmere fiber production in goat.

Gene name	Chromosome	Exon	Function	Founder breeds/population	References
*LHX2*	11	5	Involved with the development of secondary hair follicles (SHF)	Inner Mongolia cashmere	[18, 111]
*FGF9*	12	3	Promotion of hair follicle regeneration after wounding	Inner Mongolia cashmere	[18, 113]
*WNT2*	4	5	Involved in hair follicle initiation	Inner Mongolia cashmere	[18, 112]
*NOTCH1*	11	34	Controlling follicular proliferation rates as well as melanocyte populations	Liaoning cashmere, goat	[12]
*FGF5*	6	3	Regulation of hair length and potentially involved with cashmere fiber traits	Cashmere breed	[114]
*IGFBP7*	6	5			
*OXTR*	22	5			
*ROCK1*	24	33			
*SGK3*	14	18	Has an important role in the development of postnatal hair follicle	Cashmere breed	[114, 115]
